# Individual Differences in Sensitivity to Visuomotor Discrepancies

**DOI:** 10.3389/fpsyg.2019.00144

**Published:** 2019-02-04

**Authors:** John Dewey, Shane Mueller

**Affiliations:** ^1^Department of Psychological Sciences, University of North Georgia, Dahlonega, GA, United States; ^2^Department of Cognitive and Learning Sciences, Michigan Technological University, Houghton, MI, United States

**Keywords:** control, sense of agency, visuomotor detection task, individual differences, motor performance

## Abstract

This study explored whether sensitivity to visuomotor discrepancies, specifically the ability to detect and respond to loss of control over a moving object, is associated with other psychological traits and abilities. College-aged adults performed a computerized tracking task which involved keeping a cursor centered on a moving target using keyboard controls. On some trials, the cursor became unresponsive to participants’ keypresses. Participants were instructed to immediately press the space bar if they noticed a loss of control. Response times (RTs) were measured. Additionally, participants completed a battery of behavioral and questionnaire-based tests with hypothesized relationships to the phenomenology of control, including measures of constructs such as locus of control, impulsiveness, need for cognition (NFC), and non-clinical schizotypy. Bivariate correlations between RTs to loss of control and high order cognitive and personality traits were not significant. However, a step-wise regression showed that better performance on the pursuit rotor task predicted faster RTs to loss of control while controlling for age, signal detection, and NFC. Results are discussed in relation to multifactorial models of the sense of agency.

## Introduction

Many everyday behaviors, such as driving automobiles and playing video games, involve controlling moving objects. Occasionally, people may suddenly lose control of these systems. For example, a car may lose traction on an icy road, and a computer mouse cursor will stop responding if it accidentally becomes unplugged. In situations like these, what individual differences might influence how quickly a person recognizes and responds to a loss of control?

The psychological literature on action planning and motor control suggests that people experience reduced control when they become aware of discrepancies between their intended actions and perceptual feedback. Thus, detecting loss of control must largely depend on predictive processes within the nervous system. We use “predictive process” as shorthand for the brain’s ability to anticipate the perceptual consequences of voluntary actions (Wolpert et al., 1995; Prinz, 1997; Hommel et al., 2001). It is well-established that the predictability of actions and their outcomes contributes to the sense of agency, i.e., the phenomenology of willfully causing something to happen (Gallagher, 2000; Haggard, 2005; Haggard and Tsakiris, 2009). For example, anticipation of the visual, proprioceptive, and kinesthetic feedback produced by voluntary body movements is one mechanism by which individuals recognize those movements as self-generated (Blakemore et al., 1999; Frith, 2005; Farrer et al., 2008).

**FIGURE 1:**
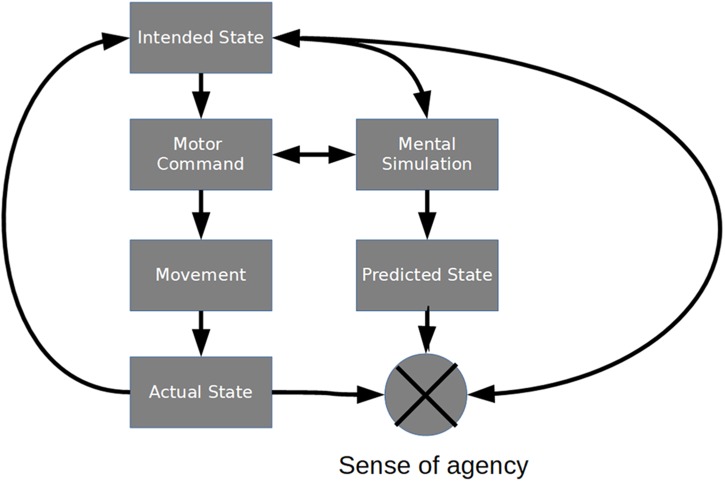
Comparator model of the sense of agency. Control involves a coupling of perceptual, motor, cognitive, and metacognitive processes. Motor actions are made in response to monitoring differences between the actual state of the system and intended (or future) goal states. A sense of agency arises from comparing the intended, predicted, and actual states of the system as motor commands are carried out. Awareness of discrepancies leads to detection of loss of control.

Similarly, when people manipulate moving objects external to the body, they may experience a loss of control if movements which were previously predictable become unpredictable. For example, judgments of control over moving objects can be modulated by adding degrees of unpredictable spatial or temporal perturbations to visual feedback (Metcalfe and Greene, 2007; Dewey et al., 2010; Dewey et al., 2014). Figure 1, which is based on comparator models of the sense of agency (Frith and Done, 1989; Frith, 2005, 2012), depicts a conceptual model of a control loop that incorporates major processes of motor control, action prediction, and monitoring. To summarize, detecting a loss of control over a moving object requires monitoring one’s own internal state (intended and executed movements) in tandem with changes to the external environment (visual or other perceptual feedback) to ensure consistency.

The goal of the present study was to explore individual differences that might be associated with sensitivity to visuomotor discrepancies, specifically the ability to detect and respond to a loss of control over a moving object. Sustained monitoring for unpredictable visuomotor discrepancies requires focused attention and activates functional networks also involved in cognitive control, specifically lateral and medial prefrontal cortices (Schnell et al., 2007). In behavioral studies, executive abilities and fine motor skills often overlap to a degree, although these effects seem to depend on the novelty and difficulty of the task (Stuhr et al., 2018). For example, some studies have found associations between manual dexterity and inhibitory control (Livesey et al., 2006; Rigoli et al., 2012). We were interested in the exploring whether sensitivity to loss of control in a visuomotor domain would be associated with cognitive abilities or trait-based personality inventories that are typically predictive over longer timescales and for more complex tasks. Because there are few published studies on individual differences in this area, we administered a diverse variety of behavioral and questionnaire-based measurements. The remainder of the introduction provides an overview of the measurements included in the study, justifications for why those measurements were included, and associated hypotheses.

To measure participants’ sensitivity to visuomotor discrepancy, we used a modified version of a task previously used to study the phenomenology of control over moving objects (Dewey et al., 2014). The task involves following a visual target on a computer screen as closely as possible using a cursor the participant controls with the keyboard (see section “Materials and Methods”). Previous testing has shown that participants experience varying degrees of control depending on the correlation between their input and the visual feedback indicating the continuously updated position of the cursor. In the present study, loss of control was implemented by breaking the link between input and feedback entirely, such that user input ceased to have any effect on the cursor. By instructing participants to respond as soon as they noticed a loss of control, we obtained accuracy and response time (RT) data.

Next, we populated a list of traits and abilities with theoretical links to the sense of agency at different levels, beginning with basic motor control. In many domains there is an association between individuals’ competence at a task and the accuracy of metacognitive judgments pertaining to that task (Kruger and Dunning, 1999; Gray et al., 2007; Ehrlinger et al., 2008). The coordinated control of hand and eye-movements is necessary to succeed at many tasks that might evoke a sense of agency, including the manipulation of moving objects. Therefore, we hypothesized that better performance on the pursuit rotor task, a measure of hand-eye coordination, would be associated with faster and more accurate detection of loss of control.

Detecting visuomotor discrepancies logically requires attentive monitoring of sensory feedback, and the ability to detect qualitative changes in that input stream. We used a task that involves watching an instrument panel for signals among noise to measure participants’ attentive monitoring capability in a task that does not incorporate prediction. We hypothesized that participants who performed better on this signal detection task would also respond faster and more accurately to visuomotor discrepancies on the loss of control task.

At a higher level of complexity, impulsiveness is a cognitive/personality/behavioral construct relating to the control of internal states, particularly inhibition of undesirable behaviors. It can be defined as a tendency toward quick, unplanned reactions to stimuli with little regard for potential negative consequences (Stanford et al., 2009). Impulsiveness is associated with risk-taking, certain maladaptive behaviors, and increased activity in emotional brain centers during reward anticipation (Baylé et al., 2003; Martins et al., 2004; Kerr et al., 2015). Self-reported impulsiveness has been associated with performance on a variety of cognitive tasks related to inhibition, including the Stroop task, stop-signal, go/no-go tasks, and anti-saccade tasks, although the magnitude of those associations is small (Aichert et al., 2012). Additionally, people with attentional deficit disorder may be poor at monitoring changes (Cohen and Shapiro, 2007). In relation to the loss of control task, impulsiveness could manifest as failing to monitor the consequences of one’s actions, perhaps due to wandering attention. Therefore, we hypothesized that if trait impulsiveness has any association with detecting loss of control, it would be in the direction of higher impulsiveness associating with slower RTs and reduced accuracy.

Schizotypy refers to an individual’s tendency toward magical thinking, perceptual abnormalities, and other symptoms associated with schizophrenia. Frith and Done (1989) proposed that some schizophrenic patients suffer from an impairment in predicting the perceptual consequences of their thoughts and actions, which leads to attribution errors. Schizophrenic populations are impaired at recognizing feedback produced by their own movements (Franck et al., 2001), and show evidence of imprecise sensory predictions and impaired associative learning (Synofzik et al., 2010; Moore et al., 2011). Schizophrenic patients are also more likely than healthy controls to depend on publicly available external cues (i.e., the appearance of a successful outcome) when judging their control over moving objects (Metcalfe et al., 2012). It is unclear whether non-pathological variation in schizotypy influences sensitivity to loss of control. In one recent study of a healthy college-aged population, individual differences in schizotypy were not significantly associated with participants’ confidence that an auditory stimulus was triggered by their own actions (Dewey and Knoblich, 2014). However, in a task more reminiscent of the present study, a high degree of schizotypy was associated with a reduced sense of control over the movements of a mouse cursor (Asai and Tanno, 2007). If self-report measures of schizotypy tap individual differences in predictive processes, then increased schizotypy might be associated with decreased accuracy and increased RTs to loss of control.

Locus of control (LOC) refers to the extent to which individuals feel in control of their own lives (Rotter, 1966). LOC is conceptualized along a continuum from external to internal. People with an internal LOC generally believe they have the power to influence events, while those with an external LOC believe external forces (chance or powerful others) exert greater influence (Levenson, 1973). LOC is often treated as a stable personality trait; however, some studies have reported shifts toward an internal LOC following interventions (Hattie et al., 1997; Hans, 2000), which would indicate that LOC is influenced by learning. If LOC is influenced by a lifetime of self-observations regarding one’s ability to predict the consequences of actions, then an internal LOC might be associated with more precise internal predictions, and thus faster and possibly more accurate responses to loss of control. On the other hand, a very strong internal LOC might also bias individuals to believe they have control even when they do not (i.e., a strong prior belief in control), which would predict slower RTs to loss of control in individuals with a more internalized LOC.

Need for cognition (NFC) is a construct reflecting an individual’s enjoyment of cognitively demanding activities, such as learning new skills or solving difficult problems (Cacioppo and Petty, 1982). It has been associated with susceptibility to cognitive bias, false memories, and personality traits such as openness to experience (Petty et al., 2009). In general, NFC relates to how much information individuals seek across a range of situations. For present purposes, we used NFC as a convenient proxy for motivation at the trait level (i.e., not governed by the situation). In relation to control, a higher NFC might be associated with more accurate monitoring of internal and external states, assuming such individuals are more likely to be attentive and motivated to perform well. Consequently, we hypothesized that a higher NFC would be associated with faster and possibly more accurate responses to loss of control.

Finally, automobile driving and videogames are two real-world applications which involve the control of moving objects. To explore whether sensitivity to visuomotor discrepancy in our loss of control task is associated with performance of these activities, later participants in the study were asked to estimate how many hours per week they spent playing videogames, and whether they had ever been involved in a serious auto accident (more than a “fender bender”). We hypothesized that spending more time on videogames would be associated with better tracking performance as well as faster and more accurate responses to loss of control, reflecting gamers’ familiarity and skill with similar visuomotor tasks. We also hypothesized that involvement in one or more serious auto accidents would be associated with slower and possibly less accurate responses to loss of control.

## Materials and Methods

### Participants

An *a priori* power analysis using G^∗^Power (Faul et al., 2009) for linear bivariate regression (one group) determined that a sample size of 105 would be sufficiently powered (1 – β = 0.80) to detect medium-sized (0.3) or stronger associations with an alpha of 0.01. However, our actual sample size was determined by how many participants we could recruit during over two academic semesters. One hundred and fifteen students from the University of North Georgia and Michigan Technological University, all with normal or corrected-to-normal vision, voluntarily participated in the study in exchange for course credit. All participants completed the questionnaires. Eighty-one of the participants additionally completed the behavioral tasks. Thus, the final sample size for most analyses of interest was 81. Due to an error during data collection, demographic data were only collected for 58 participants (mean age = 19.14; 31 females; age range = 18–22). All procedures were approved by the institutional review boards at both institutions.

### Procedure and Stimuli

#### Questionnaires

In the first phase of the study, participants completed several self-report questionnaires. These were completed on site in either the first or second author’s lab space and administered on a desktop PC using Google Forms. Participants followed links on a web page to reach the questionnaires and could complete them in any order.

The Barratt Impulsiveness Scale Version 11 was administered to measure impulsiveness. The BIS-11 is the current gold-standard for self-report measures of impulsiveness, and it distinguishes three factors: Attentional (lack of focus), Motor (acting without thinking), and Non-planning (lack of forethought) (Patton et al., 1995; Stanford et al., 2009).

The Magical Ideation Scale and Perceptual Abberation Scale were administered to measure schizotypy. The Magical Ideation Scale measures belief in non-valid forms of causation, such as extrasensory perception, while the Perceptual Abberation Scale measures disturbances in perception, particularly in relation to body-image (Chapman et al., 1978; Eckblad and Chapman, 1983).

The Levenson Multidimensional Locus of Control Scales (1973) were administered to measure trait-based LOC. This scale distinguishes three factors: Internality, Powerful Others, and Chance. For present purposes, we only considered the first factor, Internality, which measures the tendency to attribute events to one’s own agency. (The latter two factors distinguish whether external influences are attributed to powerful others, such as parents or political leaders, or chance events.)

Need for cognition was operationalized using a short-form developed by Cacioppo et al. (1984). This measures participants’ enjoyment of challenging problems and thinking.

Finally, some participants completed a customized demographic and historiographic questionnaire, which included the following questions:

Q1 What is your gender?
Q2 What is your age?
Q3 How many hours per week do you play videogames?
Q4 Have you ever been involved in a serious accident while driving (more than a “fender bender”), regardless of whether you were at fault?

During the first semester of data collection, participants would complete the various questionnaires on the first day of the study and were asked to return on a later date for the behavioral phase of the study. Due to high attrition, this protocol was modified the following semester so that participants completed the questionnaires and behavioral tasks in a single session. The final sample included 13 participants who completed the two phases on two separate days, and 68 who completed both phases on the same day. Following this change, participants proceeded to the behavioral phase immediately after completing the questionnaires.

#### Behavioral Tasks

In the second phase of the study, participants completed three behavioral tasks: the loss of control task, the pursuit rotor task, and the probability monitor task. Task order was partially counterbalanced. Approximately half (40) the participants completed the loss of control task first, while half completed the pursuit rotor and probability monitor task (in randomized order) before the loss of control task.

A modified version of a task previously developed by Dewey et al. (2014) was used to operationalize participants’ sensitivity to visuomotor discrepancies (the most substantive changes involved adjustments to numerical parameters controlling the behavior of the moving target, as well as the removal of the probe for collecting judgments of control at the end of each trial). The stimuli were programmed in MATLAB using display functions provided by the psychophysics toolbox (Brainard, 1997). The task involved keeping a cursor centered on a moving target using keyboard controls. Each trial began with the user-controlled cursor (a solid red circle) and target (a black outline of a circle) at the center of the display. After one second, the target began moving left and right in an unpredictable fashion, changing speed and direction at random intervals. Participants used the left and right keyboard arrows to accelerate the cursor leftward or rightward, with the goal of staying as close to the target as possible. There was no simulation of friction, so once in motion the cursor would continue moving at a constant rate unless the participant intervened.

On half of the trials, participants began to lose control of the cursor at a randomly determined point. The loss of control was constrained to occur at a point between 20 and 80% of the maximum duration of a trial (16.67 s), so it never happened at the very start or end of a trial. To avoid an obvious and abrupt transition from full control to no control, the proportion of the cursor’s motion controlled by the participant’s keyboard input decreased from 100 to 0% in a linear fashion over a span of 1000 ms. As control decreased, the cursor’s motion was increasingly determined by the combination of previous momentum and noisy perturbations generated by adding together three randomly phased sine waves. Participants were instructed to press the spacebar as quickly as possible if they noticed that they no longer had control over the cursor. RTs were measured starting from the beginning of the loss of control; i.e., the first frame with noisy perturbations. Pressing the space bar at any point immediately ended the trial. On trials where the space bar was not pressed, the trial would end after 16.67 s. Participants completed 40 trials of the loss of control task.

The pursuit rotor task was administered to measure participants’ hand-eye coordination in the absence of visuomotor discrepancy. This task involves tracking a moving disk as accurately as possible as it travels a circular path. A computerized version of the pursuit rotor task using a mouse-controlled cursor was administered using the Psychology Experiment Building Language (PEBL) (Mueller and Piper, 2014). Participants completed four 15-s trials. The performance metric for this task was the proportion of time on target.

Finally, the probability monitor task was administered to measure participants’ sensitivity to signals in noisy visual input. Participants were tasked with monitoring three dials and responding when one showed a signal (a bias toward the left or right side). The probability monitor task was administered using PEBL (Mueller and Piper, 2014). Participants completed 11 trials averaging about 17 s each. The speed and accuracy (hits and false alarm rates) of participants’ responses were recorded.

## Results

The loss of control task measured participants’ ability to detect and respond to a visuomotor discrepancy. There were three dependent measures associated with this task: (1) time on target, defined as the proportion of each trial that the user-controlled cursor at least partially overlapped with the moving target prior to the loss of control, (2) the accuracy of participants’ responses to loss of control; and (3) the RTs to loss of control. Descriptive statistics for those three dependent measures are summarized at the bottom of Table 1. The mean for the main dependent variable of interest, RT to loss of control, was nearly 4 s (*SD* = 1.28). Slow RTs can most likely be attributed to a combination of factors related to how the stimulus was programmed. First, input from the participant was not necessarily continuous. Brief intervals (typically < 1 s) could occur when no input was required to keep the cursor on target. Second, because the moving cursor kept its momentum without simulating friction, participants’ control could have been obscured. For example, if the cursor was moving at high velocity in the rightward direction and the left key was pressed, this would not instantly change the cursor’s direction. Instead, the cursor would slow down (linearly), stop, then begin accelerating leftward. Thus, although keyboard latency was minimal, there would often be a lag between participants’ first attempt to move in a particular direction and achievement of that result. The 13 participants who completed the questionnaire and behavioral tasks across 2 days in the first semester of data collection had a mean RT of 3.43 s (*SD* = 0.42), and the 68 participants who completed the study in a single day in the second semester of data collection had a mean RT of 3.87 s (*SD* = 1.37). Importantly, there was considerable variability across participants in all three measures, and the accuracy of responses, while high (∼83%), was not at ceiling.

**Table 1:** Correlations (Spearman’s rho) and descriptive statistics for loss of control task (*n* = 81).

	Loss of control task		
	
	Time on target	RT (s)	Accuracy
Age	0.20	-0.05	0.04
Gender (m = 1)	0.48^*^	-0.17	0.07
Videogames	0.39^*^	-0.03	0.09
Auto accidents	-0.04	-0.11	0.07
Pursuit rotor	0.64^*^	-0.30^†^	0.19
Prob. Mon (acc)	0.26	-0.11	0.20
Prob. Mon (rt)	-0.23	0.09	0.04
LOC (I)	-0.01	0.00	-0.05
NFC	0.24	0.02	-0.05
BIS (A)	-0.15	-0.02	-0.18
BIS (M)	0.03	-0.11	-0.02
BIS (N)	-0.15	-0.09	-0.04
PA	-0.04	-0.10	-0.21
MI	-0.15	0.00	-0.15
*M*	0.81	3.80	0.83
*SD*	0.07	1.28	0.19


^†^*p < 0.1; *^∗^p* < 0.05 (corrected for false discovery).*

To assess the reliability of the loss of control task within participants, a split-half reliability analysis was performed on the RT data. This analysis only included trials where participants noticed a loss of control (i.e., “hits”). Mean RTs for half of each participant’s trials, randomly selected, were correlated the mean RTs from the other half of trials. Spearman–Brown correction was applied to the correlations. The loss of control task was reliable with ρ = 0.85.

Our main research question was whether performance on the loss of control task was associated with the other measured variables. To account for deviations from normality, Spearman’s rho is reported as a non-parametric measure of correlation magnitudes. A corrected alpha level of 0.05 was used for all inferential statistics. *p*-Values were adjusted using the “fdr” method of Benjamini and Hochberg (1995) to control the false discovery rate.

Bivariate correlations between the loss of control task and other measured variables are summarized in Table 1. (Associations between the questionnaires were non-central to our hypotheses and are reported in the Supplementary Materials.) Time on target (i.e., tracking performance prior to loss of control) in the loss of control task was positively associated with maleness, *r_s_* = 0.48, hours of videogames played per week, *r_s_* = 0.39, and with performance on the pursuit rotor task, *r_s_* = 0.64. No other variables were significantly related to performance on the loss of control task, although there was a marginally significant negative association between the pursuit rotor task and RTs to loss of control, *r_s_* = -0.304, suggesting that participants with better hand-eye coordination may respond faster to loss of control.

Next, to identify the most promising subset of variables for predicting sensitivity to visuomotor discrepancy, the same variables indicated in the rows of Table 1 were submitted to a linear stepwise forward regression analysis, using RTs to loss of control as the criterion variable. Since none of the simple bivariate correlations for this criterion were statistically significant, we used a liberal stepping criterion to get the model started. The criterion for adding predictor variables to the regression model was a *p*-value less than 0.08, while the criterion for removal of variables was a *p*-value greater than 0.1. As shown in Table 2, the three statistically significant Beta weights were for age (older age indicating slower RTs), the pursuit rotor task (better tracking performance indicating faster RTs), and the probability monitor task (more accurate signal detection indicating slower RTs). NFC showed a marginally significant association with RTs to loss of control (higher NFC indicating faster RTs).

**Table 2:** Standardized coefficients for stepwise forward regression predicting response times to loss of control.

	Standardized beta	*p*
Age	0.41	0.009
Pursuit rotor	-0.38	0.02
Prob mon. (acc)	0.33	0.03
NFC	-0.27	0.07


Finally, we used an exploratory factor analysis to identify underlying relationships among the measures. The analysis was performed using the ‘fa’ function within the psych library (Revelle, 2017), version 1.6.8, of Version 3.3.1 of the R statistical computing language (R Core Team, 2016). An initial principal components analysis using eigen decomposition indicated that four factors were likely sufficient to account for systematic covariance in the data. As a cutoff, new factors were added until any new factor that was added accounted for less than 5% of the variance.

The factor analysis used the minimum residuals optimization method with oblimin translation, which produced the inter-factor correlations shown in Table 3. Correlations between the factors explain why some measures loaded onto more than one factor. The factor loadings are summarized in Table 4. Results indicated that factor F1 was related to the BIS-11 impulsiveness scales and negatively related to NFC and LOC (I). A second factor, F2, was most strongly related to performance on the pursuit rotor and loss of control tasks. This further supports the link between performance on the pursuit rotor and loss of control tasks. The third factor, F3, was related to PA and MI (the schizotypy subscales), and the fourth factor, F4, was most clearly related to the probability monitoring task and videogame experience.

**Table 3:** Correlations among the factors in the exploratory factor analysis and sum of squared (SS) loadings.

	F1	F2	F3	F4
F1	1			
F2	-0.10	1		
F3	-0.18	-0.08	1	
F4	-0.06	0.23	-0.15	1
*SS loadings*	2.08	1.91	1.77	1.15
*Proportion Var*	0.14	0.13	0.12	0.08
*Cumulative Var*	0.14	0.27	0.38	0.46


**Table 4:** Factor scores for the exploratory factor analysis.

Measure	F1	F2	F3	F4
Videogames	-	0.26	-0.21	**0.57**
Auto accidents	-	-	**0.21**	0.12
Loss of control (time on target)	**-**	**1.00**	-	-
Loss of control (accuracy)	**-**	**0.19**	-0.18	-
Loss of control (response time)	**-**0.11	**-0.49**	-	**-**
Pursuit rotor	-	**0.56**	-	0.46
Prob. mon (acc)	-	0.17	-	**0.25**
Prob. mon (rt)	-	-0.34	-	**0.54**
LOC (I)	**-0.46**	**-**0.14	0.14	0.39
NFC	**-0.55**	**-**0.15	-	-
BIS (A)	**0.54**	-	0.25	
BIS (M)	**0.59**	-	0.12	
BIS (N)	**0.91**	-	-	
PA	0.13	-	**0.86**	
MI	-	-	**0.88**	


*Values smaller than ±0.1 are removed and shown as dashes. The greatest factor loading for each measure is indicated with bold script.*

## Discussion

The goal of this study was to explore potential correlates of sensitivity to visuomotor discrepancy. We hypothesized several potential associations between this ability and a variety of behavioral and psychological traits. Although we found evidence that better hand-eye coordination is associated with faster recognition of loss of control, other hypothesized relationships between stable psychological traits and sensitivity to visuomotor discrepancy were not supported.

Analysis of the bivariate correlations summarized in Table 1 indicated that hand-eye coordination, operationalized with the pursuit rotor task, was marginally positively associated with sensitivity to loss of control, but none of the trait-based questionnaires were (see Table 1), and neither was performance on a non-motor monitoring task (the probability monitor). An important caveat to this finding is that, due to participant attrition, our final sample size of 81 was underpowered to detect correlations weaker than about 0.3 (i.e., medium-sized effects). In any case, our results suggest that associations between sensitivity to visuomotor discrepancy and stable psychological traits such as impulsivity, schizotypy, and LOC are not large in magnitude for the population that was studied.

Although none of the variables studied was significantly associated with sensitivity to visuomotor discrepancy in isolation, a stepwise forward regression analysis indicated that a subset of the variables (age, and performance on the pursuit rotor and probability monitor tasks) taken together did significantly predict RTs to loss of control (see Table 2). Age was positively associated with RTs, indicating that older participants responded to loss of control more slowly while accounting for the other predictor variables. Although age-related differences in reaction times are a common finding in psychology, our participants were all between 18 and 22 years old, so it is unclear what drove this result. Performance on the pursuit rotor was negatively associated with RTs, indicating that people with better hand-eye coordination responded to loss of control more quickly. A close relationship between these two tasks was further confirmed by an exploratory factor analysis, which showed that time on target in the pursuit rotor task and RTs to the loss of control task substantially loaded onto the same factor. This makes intuitive sense, as successfully tracking a target recruits the same predictive motor control processes which are thought to give rise to the sense of agency in the first place. Conversely, accuracy on the probability monitor task was positively associated with RTs to loss of control (once age and PR tracking were taken into account). This could be indicative of a speed/accuracy tradeoff style across participants, as taking plenty of time to ensure an accurate response would be a good strategy for the probability monitor task, but not for speedy detection of loss of control. Lastly, our exploratory factor analysis revealed four moderately coherent factors which generally supports the idea of systematic individual differences in the component abilities for control.

As reviewed in the introduction, previous research suggests predictive processes are foundational to the sense of control over moving objects, and to the sense of agency more generally. However, there is an emerging consensus that the comparator model (see Figure 1) of agency, despite its usefulness, is overly simplistic. The sense of agency depends not only on proximal factors, such as the moment-to-moment predictability of a stimulus, but also from more distal factors, such as the achievement of goals (Metcalfe and Greene, 2007; Pacherie, 2008; Dewey et al., 2010, 2014). Even at short times scales, the sense of agency seems to be influenced by a combination of predictive sensorimotor processes and *post hoc* inferences (Sato, 2009; Takahata et al., 2012; Kumar and Srinivasan, 2014). Synofzik et al. (2008, 2013) proposed a model of agency in which different cues (predictive and inferential) to self-agency are combined and weighted according to their perceived reliability. For example, schizophrenic individuals seem to rely less on predictive cues and more on publicly available information (Metcalfe et al., 2012), presumably because their sensory predictions are less precise compared to healthy control populations (Franck et al., 2001; Asai et al., 2008; Synofzik et al., 2010). In light of this proposal, a straightforward explanation of our results is that most participants relied heavily on predictive sensorimotor cues to detect a loss of control, and contrary to our original hypotheses, the reliability of these cues did not systematically covary with stable personality traits such as impulsivity, LOC, NFC, or non-clinical schizotypy.

A future direction for this line of work is exploring individual differences in the sense of agency when sensorimotor cues are ambiguous or unavailable. If cues to self-agency are weighted per their reliability, then reducing the usefulness of sensorimotor predictions might open the door to other factors exerting a greater influence. One variable that can affect this is control scheme, i.e., user interface, a topic that is receiving attention in the field of human computer interactions (Lakshika and Barlow, 2017). Distracting attention or forcing participants to multitask may be another way to reduce the reliability of sensorimotor cues. There may also be additional personality variables beyond those tested in the present study that could be related to sense of agency. For example, in some situations depressed individuals known to be more accurate than non-depressed controls at judging contingencies between their responses and other events (Alloy and Abramson, 1979). Whether depressed individuals would also be less likely to overestimate their control over moving objects is, to our knowledge, an open question.

Another possibility would be to re-investigate some of the hypothesized relationships from the present study using more sensitive measures. For example, we used short survey forms to measure trait Impulsivity (using the BIS-11) and NFC, a decision that was made to limit the duration of our experiment to an hour. However, transient attentional and motivational influences on behavior could be measured precisely using behavioral tasks. Another limitation of the present work is that we only had time to collect data from a small number of trials for the behavioral tasks (e.g., 40 trials/participant for the loss of control task) and, with limited data to work with, included all trials for analysis without screening for outliers. Measuring loss of control with enough precision to compute d prime and to perform better quality control on the data would likely require 100s of trials, but would provide a richer view of participants’ behavior.

The literature on human control suggests several distinct abilities or cognitive components that may contribute to the phenomenology of action. Identifying individual differences in related abilities, including the ability to detect loss of control over moving objects, could have both theoretical and practical implications. On the theoretical side, this research addresses the degree to which agency judgments depend on situational vs. participant characteristics. From an applied perspective, documenting associations between sensitivity to loss of control and stable psychological traits might be useful for predicting performance in task domains that involve monitoring control systems. The results of the present study suggest the ability to monitor and respond to visuomotor discrepancies, which is foundational to the sense of agency, can be predicted from an individual’s hand-eye coordination, but not from stable psychological traits such as LOC, impulsivity, NFC, or impulsivity.

## Data Availability

The data that support the findings of this study are publicly available on the Open Science Framework and can be accessed at: osf.io/kt326.

## Ethics Statement

This study was carried out in accordance with the recommendations of the institutional review boards at the Michigan Technological University and the University of North Georgia with written informed consent from all subjects. All subjects gave written informed consent in accordance with the Declaration of Helsinki. The protocol was approved by the institutional review boards at the Michigan Technological University and the University of North Georgia.

## Author Contributions

JD proposed the study, administered the experiments, analyzed the data, and wrote the manuscript. SM collaborated in the design, administration, and data analysis.

## Conflict of Interest Statement

The authors declare that the research was conducted in the absence of any commercial or financial relationships that could be construed as a potential conflict of interest.
