# Supplementary material for: Individual Differences in Sensitivity to Visuomotor Discrepancies
Source: Front Psychol. 2019 Feb 4;10:144. doi: 10.3389/fpsyg.2019.00144 (PMC6369185; doi:10.3389/fpsyg.2019.00144)
Supplement: Supplementary file 1 [file Table_1.DOCX]

Supplementary Table

*Correlations and descriptive statistics among questionnaire-based measures (n = 115)*

|  | 1 | 2 | 3 | 4 | 5 | 6 | 7 |
| --- | --- | --- | --- | --- | --- | --- | --- |
| 1. LOC (I) | - | .21 | -.32* | -.28* | -.36* | -.06 | -.06 |
| 2. NFC |  | - | -.27* | -.37* | -.53* | -.03 | .04 |
| 3. BIS (A) |  |  | - | .45* | .52* | .27* | .20 |
| 4. BIS (M) |  |  |  | - | .66* | .15 | .10 |
| 5. BIS (N) |  |  |  |  | - | .12 | .13 |
| 6. PA |  |  |  |  |  | - | .74* |
| 7. MI |  |  |  |  |  |  | - |
| *M* | 33.4 | 61.97 | 18.82 | 23.18 | 21.84 | 6.02 | 7.66 |
| *SD* | 6.24 | 10.55 | 3.83 | 4.31 | 4.73 | 5.62 | 5.89 |
| *Range* | 19-45 | 39-83 | 9-27 | 16-38 | 11-33 | 0-29 | 0-24 |

**p* < .05 (corrected for false discovery)
